# Descriptive, injunctive, or the synergy of both? Experimenting normative information on behavioral changes under the COVID-19 pandemic

**DOI:** 10.3389/fpsyg.2022.1015742

**Published:** 2022-12-22

**Authors:** Isamu Okada, Itaru Yanagi, Yoshiaki Kubo, Hirokazu Kikuchi

**Affiliations:** ^1^Graduate School of International Development, Nagoya University, Nagoya, Japan; ^2^School of Law, Ritsumeikan University, Kyoto, Japan; ^3^Department of Urban Studies, School of Policy Studies, Kwansei Gakuin University, Sanda, Hyogo, Japan; ^4^Department of Law, Politics, and International Relations, Faculty of Humanities and Social Sciences, University of the Ryukyus, Nishihara, Okinawa, Japan; ^5^Research Institute for Humanity and Nature, Kyoto, Japan; ^6^Department of East Asian Languages and Cultures, Hamilton Lugar School of Global and International Studies, Indiana University, Bloomington, IN, USA; ^7^Program on U.S.-Japan Relations, Weatherhead Center for International Affairs, Harvard University, Harvard University, Cambridge, USA; ^8^Area Studies Center, Institute of Developing Economies, Japan External Trade Organization, Chiba, Japan

**Keywords:** COVID-19, behavioral change, social norms, synergistic effects, Japan

## Abstract

**Backgrounds:**

The effectiveness of citizens’ behavioral changes to prevent the spread of SARS-CoV-2, such as avoiding large social events, relies on science communication from policymakers and collective action among peer citizens. Extant studies recognize the potential effects of information stimuli on citizens’ behavioral changes, including what epidemiological experts request (*injunctive information*) and what surrounding people behave (*descriptive information*). Yet, they have insufficiently assessed the co-occurrence and possible interaction of multiple information stimuli.

**Methods:**

1,819 Japanese citizens aged 18 or over were recruited for an experimental survey during March 1–3, 2021 and asked their views on a hypothetical wedding attendance in Japan while being exposed to randomly assigned normative information stimuli. Their willingness to attend a wedding asked before and after the intervention was measured. Infection risk perception was also asked as a mediating variable.

**Results:**

Findings suggest the constant supremacy of descriptive information and no synergistic effects in the interaction of multiple information stimuli. We also report that the effects of injunctive and descriptive information vary according to participants’ risk perception, age, and trust in experts.

**Conclusion:**

Our experimental test enables a systematic assessment of multiple normative information and confirms the primacy of descriptive information as the main driver of behavioral change. Communication by medical experts has limitations but is still effective in specific categories of the population.

## 1. Introduction

The COVID-19 pandemic has demanded citizens’ behavioral changes worldwide for an extended period. Since the early stage, studies have shown that behavioral changes decrease the effective reproduction number of SARS-CoV-2 (Kraemer et al., 2020; Perski et al., 2021) and argued that social and behavioral scientists have rooms to provide answers on this aspect (Bonell et al., 2020; Van Bavel et al., 2020; Perra, 2021). The ongoing debate by social scientists is rich, diverging perspectives suggest different causal mechanisms. On the one hand, behavioral changes are regarded as the product of effective communication by epidemiologists and government leaders (Lunn et al., 2020a,b; Casoria et al., 2021; Galbiati et al., 2021; Bokemper et al., 2022; Garett et al., 2022). On the other, especially in countries with soft lockdown, the effectiveness of social distancing is claimed to rely on the sensitivity to expected behavior among peer citizens (Cato et al., 2020; Yong and Choy, 2021; Čavojová et al., 2022; Paniagua, 2022). While the two mechanisms may work in tandem or interactively in the actual context, how precisely they affect the citizens’ behavior has remained unclear.

Addressing the difference between the two mechanisms, social psychologists have focused on injunctive norm—what is socially approved or disapproved—and descriptive norm—how people behave—each corresponding to the above-mentioned causal mechanisms (Young and Goldstein, 2021). Studies so far have acknowledged the robust effects of both norms in shaping behavioral changes of citizens under the COVID-19 pandemic (Chi et al., 2021; Cucchiarini et al., 2021; Gouin et al., 2021; Irawan et al., 2021; Kang et al., 2021). Yet, so far, the scholarly attention has stopped short of addressing the co-occurrence and interaction of the two social norms. In the real-world context, we live in an environment where information stimuli conveying injunctive and descriptive norms are multifold and simultaneously at play.

Do multiple normative information stimuli interactively lead to citizens’ behavioral change? Or do they work separately and generate additive effects on citizens’ behavior? To answer these questions, we employed an experimental survey conducted in Japan during March 1–3, 2021. As an analytical strategy, we focus on the case of marriage ceremony attendance. We consider wedding attendance to be the most ambivalent case (See Supplementary File 1 for further details). It typically employs eating and drinking in close contact with many people, which heightens the infection risk and is hence considered an infection-spreading event. But the complete avoidance of such a fundamental life event where families and friends bond and reconfigure their relationships is also socially questionable. Moreover, an invitation to a wedding is not very frequent on average. Therefore, people may have very few chances to experience the ceremony even a year after the outbreak of the COVID-19 pandemic.

## 2. Theory and hypothesis

### 2.1. Theoretical assumptions

Our theoretical framework zeros in on the interaction of injunctive and descriptive information and the mechanism in which information stimuli mold behavioral changes. Table 1 depicts the essential parts of our theoretical assumptions.

**Table 1:** Theoretical assumptions.

	Injunctive and descriptive information are…	
	Aligned	Not aligned
Additive effects	*Separate effect* of either or both normative information	Supremacy of descriptive information
Synergistic effects	*Interactive effect,* reinforcing the decision to the aligned direction and/or updating the infection risk perception	Not applicable

In the aligned interaction of injunctive and descriptive information, we should distinguish synergistic effects from additive effects. Scholars have confirmed that an aligned interaction of injunctive and descriptive norms has a more substantial influence on the cases of alcohol consumption by college students (Cialdini et al., 2006; Lee et al., 2007; Rimal and Real, 2005), environmental conservation (Göckeritz et al., 2010), and counterproductive work behaviors (Jacobson et al., 2020). These studies, however, did not clearly distinguish two possible meanings of the interaction. On the one hand, when both injunctive and descriptive information suggest the avoidance of a mass gathering, each information stimulus may work in independent and distinctive ways, and the outcome is predicted as a sum of the two (additive effects). On the other, people may change behavior accordingly to a degree higher than what each information stimulus would achieve in total if one information stimulus reinforces the effect of the other (synergistic effects).

This suggests that the theoretical understanding of the causal mechanism requires more nuance, particularly when applied to behavioral changes under the pandemic. Conflicting social goals between the maximum containment of infection spread and the minimum externalities to people’s freedom create a high level of uncertainty as to the optimal social behavior. A few pieces of literature argue that high uncertainty by itself reduces mobility (Huynh, 2020; Cato et al., 2021; Kishishita et al., 2022), but in practical situations, the government and epidemiologists provide injunctive information to mitigate the uncertainty and guide people’s behavior. In transmitting information, citizens also have chances to witness descriptive information, i.e., how other citizens behave. Thus, injunctive and descriptive information may not only work as separate and independent stimuli but also increase or decrease the credibility of each other.

A synergistic effect posits that injunctive and descriptive information stimuli reinforce the credibility of each other by updating infection risk perception or normative belief. Ample evidence suggests infection risk perception is a robust predictor of behavioral changes under the COVID-19 pandemic (Bruine de Bruin and Bennett, 2020; Parady et al., 2020; Wise et al., 2020; Jørgensen et al., 2021). A few observational studies have suggested that the effects of perceived social norms on behavioral changes are moderated by infection risk perception (Kittel et al., 2021; Savadori and Lauriola, 2021, 2022). Yet they do not consider the interaction of injunctive and descriptive norms, holding infection risk perception as a mediator.

When injunctive and descriptive information are not aligned, robust evidence indicates that descriptive norms make people focus their attention on the environment and hence affect behavioral decisions stronger than injunctive norms when the two norms contradict each other (Cialdini et al., 1990; Cialdini, 2003). Hence, we assume an additive effect. In other words, people receive each information stimulus separately, compare them, and then make judgments.

Furthermore, normative information may work through multiple processes, such as updating infection risk perception (Kittel et al., 2021; Savadori and Lauriola, 2021, 2022), or through other mechanisms such as avoiding negative evaluation by others. Other factors may also moderate the interactive effects of injunctive and descriptive information stimuli. We focus on three aspects: trust, self-deception, and age. First, the effect of information stimuli on behavioral changes may depend on the citizen’s trust in the information source. Existing studies have reported that trusts in governments and medical experts affect behavioral changes in the case of the current pandemic (Gotanda et al., 2021; Pagliaro et al., 2021). Trust in the actors mentioned in the vignettes may also influence the reception of normative information stimuli. Second, the citizen’s susceptibility to social desirability bias may moderate the effect of information stimuli throughout the process, from information reception to decision-making (Kaczmarek and Gas, 2021; Kaufmann et al., 2022). And finally, as World Health Organization (2021) argues, younger populations may be more risk-taking than elders.

### 2.2. Hypotheses and exploratory questions

We have pre-registered hypotheses, but we decided to focus on some hypotheses and add three exploratory questions to clarify our contribution to the rapidly evolving literature.^1^

We first test pre-registered hypotheses on the individual effects of injunctive and descriptive information. Our basic assumption is that an invitation to a wedding pressures people to decide on an ambivalent question—whether to avoid infection risk or join a socially meaningful collective action. The government and medical experts provide injunctive information by suggesting the infection risk and appropriate behavior. In the case of Japan, the law bestows prefectural governors the authority to design and implement behavioral guidance. Medical experts usually assist governors by joining prefectural advisory boards. Meanwhile, we also assume the effect of descriptive information, namely what other people invited to the marriage ceremony do. Although prospect theory assumes people to be more risk-averse than risk-taking (Kahneman and Tversky, 1979), we assume that the information stimulus promoting attendance can also draw behavioral changes accordingly because infection risk is uncertain and competing information stimuli are involved in the scenario. We derive the following set of hypotheses from these assumptions.

*Hypothesis 1A*: People would choose to attend a wedding under the COVID-19 pandemic when they know their friends are attending the ceremony.

*Hypothesis 1B*: People would choose not to attend a wedding under the COVID-19 pandemic when they know their friends are not attending the ceremony.

*Hypothesis 2A*: People would choose to attend a wedding under the COVID-19 pandemic when they know that medical experts and the prefectural governor announced the low infection risk of attending the ceremony.

*Hypothesis 2B*: People would choose not to attend a wedding under the COVID-19 pandemic when they know that medical experts and the prefectural governor announced the high infection risk of attending the ceremony.

As discussed in a previous subsection, descriptive and injunctive information interaction may produce additive or synergistic effects. This conceptualization was originally not included in the pre-registration. A synergistic effect presupposes that injunctive information by the government and medical experts and descriptive information about other friends are mutually reinforcing. In contrast, an additive effect assumes separate and independent effects of each information stimuli.

When injunctive and descriptive information are not aligned, we presume that the descriptive information outweighs the injunctive one. Scholarly works in social psychology argue that injunctive information usually requires more complex cognitive processing than mimicking a clear and observable example in others’ behaviors (Cialdini et al., 1990; Cialdini, 2003). This assumption follows our pre-registration. We further explore if other conditions, such as trust in government and medical experts, self-deception, age, and living in the infection-spreading area, moderate the interactive effects of multiple information stimuli.

*Hypothesis 3*: When information about friends attending or absenting a wedding and official instructions by the prefectural governor and medical experts contradict each other, people will follow what friends’ information indicates rather than what official instructions suggest.

*Exploratory Question 1*: Do people agree more with the instruction by the prefectural government and medical experts when they know other friends are taking aligned behavior (the synergistic effect)? Or, do they take each information stimulus separately and make their decision as assumed by the sum of individual effects (the additive effect)?

*Exploratory Question 2*: Do conditions such as trust in government and medical experts, self-deception, age, and living in the infection-spreading area, moderate the interactive effects of multiple information stimuli?

One approach to clarify the causal mechanism is to check if infection risk perception mediates the effects of injunctive and descriptive information. We attempt to answer this question by causal mediation, i.e., information stimuli update each individual’s perception of infection risk, and the updated risk perception defines changes in their behavior.

*Hypothesis 4*: People’s perception of infection risk mediates the effect of information about friends and official instructions.

## 3. Materials and methods

### 3.1. Survey

We collected data through an online survey conducted on March 1–3, 2021. Registered monitors of Rakuten Insight, Inc., were recruited by quota sampling to be nationally representative of gender, age, and prefectures. When we fielded the survey, four prefectures, namely Tokyo, Chiba, Saitama, and Kanagawa, were under emergency declarations, a “soft lockdown” which kept citizens alert and restricted some economic activities—not apparently including weddings. Six other prefectures lifted the same declarations a week before the survey. We report no significant effect heterogeneity across prefectures of distinctive situations (See “Results” below). The remaining 37 prefectures did not experience emergency declarations around the time of the survey. The COVID-19 vaccination was still only available for healthcare workers, and we excluded the professional group from our sample. We used Qualtrics to design the questionnaire.

As shown in Table 2, our sample size is 1,819. We excluded four groups of people from our sample: (1) healthcare workers, healthcare officials, journalists, and those working in survey firms; (2) those who did not live in Japan at the time of the survey; (3) those who had attended a marriage ceremony since March 2020 and/or had a plan to attend one shortly, and; (4) those who failed to pass the attention check.

**Table 2:** The flow of questionnaire and exclusion.

Monitors invited from Rakuten Insight, Inc.		*n* = 4,188
**Prior consent and attention check**: While asking for prior consent, we requested participants to mark both “read very well” and “read very carefully” to the following questions. We excluded 2,010 respondents who did not agree to participate or failed to mark both options.		
**Screening 1**: We excluded 131 healthcare workers, healthcare officials, journalists, and those working in survey firms.		
**Screening 2**: We excluded 225 respondents who had attended a marriage ceremony since March 2020 or would attend soon.		
**Screening 3**: We excluded three respondents not living in Japan.		
**Sample population**		*n* = 1,819
**A set of questions**, including basic attributes, infection risk perception, perception of marriage ceremonies, etc.		
**Randomized vignette (the combination of two sentences)**		
	Group 1 (baseline): a + d (No expert information + *Friends Do not Know*)	*n* = 199
	Group 2 (treatment): a + e (No expert information + *Friends Yes*)	*n* = 201
	Group 3 (treatment): a + f (No expert information + *Friends No*)	*n* = 203
	Group 4 (treatment): b + d (*Experts No* + *Friends Do not Know*)	*n* = 201
	Group 5 (treatment): c + d (*Experts Yes* + *Friends Do not Know*)	*n* = 204
	Group 6 (treatment): b + e (*Experts No* + *Friends Yes*)	*n* = 205
	Group 7 (treatment): c + e (*Experts Yes* + *Friends Yes*)	*n* = 202
	Group 8 (treatment): b + f (*Experts No* + *Friends No*)	*n* = 203
	Group 9 (treatment): c + f (*Experts Yes* + *Friends No*)	*n* = 201
**Dependent variables and other questions**, including infection risk perception, COVID-19 experience, etc.		

Vignette compositions are explained in the following subsection. No attrition is reported after the screening.

### 3.2. Experiment

We created the following vignette and randomly assigned two sentences in the middle. As shown in Table 2, the 3 × 3 combination of sentences creates nine scenarios, one of which was randomly exposed to each survey participant. Our scenarios explicitly associated the instruction of medical experts with a press conference organized by the prefectural governor to avoid confusion due to heterogeneous opinions among medical experts in Japan. We tried assimilating the vignette and sentences to what is usually observed in the real-world context. For instance, our injunctive information stating positive about wedding attendance stresses the potential infection risk and does not directly suggest one attend an event since it seems too unnatural. Following classic works by Asch (1955) and others, we expected that congruent attitudes of more than three other people could yield substantive influence as descriptive information. We explicitly mentioned that the wedding venue is in the same prefecture since the national and prefectural governments usually requested to refrain from moving to another prefecture if any emergency declarations are issued in either or both prefectures. At the end of the vignette, we added a message regarding the instruction by affiliated organizations since it can be an impactful condition that possibly generates heterogeneous and unmeasurable effects among our sample if we do not control it.

Please let us know what you would think if something like this happens. Please read it carefully since we will check if you understood it correctly.

You received an invitation to a wedding from a person who helped you a lot a few years ago. Although the infection of novel coronavirus continues, the person wishes to organize a face-to-face ceremony since they have been preparing it for a long time. The venue is the prefecture you live in, and anyone living in other prefectures will not be invited.

<First randomized sentence>

(a) (no information)

(b) In the meantime, you watched a press conference organized by the prefectural governor in which a medical expert says that an event with many people eating and drinking together involves a low risk of infection since Japanese preemptive measures are effective.

(c) In the meantime, you watched a press conference organized by the prefectural governor in which a medical expert says that you should avoid an event that involves many people eating and drinking together because of a high risk of infection regardless of preemptive measures.

<Second randomized sentence>

When you asked four close friends who also received the invitation, you found that

(d) all of them have not decided whether to attend the wedding or not.

(e) all of them will attend the wedding.

(f) all of them will not attend the wedding

There is no explicit instruction from the organization you are affiliated with (company, school, etc.)

Both the factual manipulation check conducted after the questions on dependent variables and the randomization check confirm that information stimuli were randomly assigned and successfully introduced (See Supplementary Files 4, 5).

### 3.3. Dependent variables and main analytical design

We asked questions assessing two sets of dependent variables. First, we measured the effect on attendance decision by taking the margin between each participant’s willingness (1–7 Likert scale) to attend weddings under the pandemic asked before the vignette (*Pre-Attendance*) and the willingness to attend the wedding shown in the vignette (*Post-Attendance*). We call this variable *Attendance Change*. We consider that this pre-post measurement increases precision in gaging treatment effects (Clifford et al., 2021). We also run models using only *Post-Attendance*, but the result remains the same. Supplementary File 2 shows the answer distribution of *Pre-Attendance*, which indicates the ambiguous likeliness of wedding attendance (mean = 4.021, SD = 1.568). See also Supplementary File 3 for the frequency distribution of *Attendance Change* (mean = −0.098, SD = 1.166).

The main OLS model includes dummy variables for groups with all four friends attending (*Friends Yes*) or not attending (*Friends No*), and the expert claiming low risk (*Experts Yes*) or high risk (*Experts No*) and their interaction terms. This analytical design examines how much each information stimulus and their combinations alter *Attendance Change* compared to the baseline (all friends not decided, no information by medical expert given).

Following (Jacobson et al., 2020) and others, we used interaction terms to gage the distinction between additive and synergistic effects. Each individual information stimulus can influence the decision to attend the wedding, which is usually called the main effect. The sum of those individual main effects constitutes the additive effects. When multiple information stimuli are exposed to survey participants, they might make the wedding attendance decision by examining information stimuli not in isolation but in synergy with other information stimuli. We tested this possibility by adding two-way interaction terms of the information stimuli exhibited concomitantly in each scenario. If the beta coefficient of the interaction term turns significant, it would support the presence of synergistic effects.

### 3.4. Causal mediation analysis

We deployed causal mediation analysis (Imai et al., 2011) to address *Hypothesis 4*, which narrows down on causal mechanism through updated infection perception. In short, the causal mediation analysis tests how much the change in the infection risk perception mediates the effects of information stimuli at the level of each individual. We used the R *mediation* package (Tingley et al., 2014). The confidence intervals are calculated with robust standard errors.

We use two variables (mediators) for causal mediation analysis. First, we created the *Perception Change* variable by taking the margin between the percentages answered to the following questions.

Risk perception before the vignette: How much percentage do you think is the chance of you getting infected by SARS-CoV-2 in a year from now?

Risk perception after the vignette: In the case of the given scenario, how much percentage do you think is the chance of you getting infected by SARS-CoV-2 in a year from now?

If the normative information stimuli affect *Attendance Change* due to the heightened or lowered infection risk perception, we would observe a significant Average Causal Mediation Effect (ACME). If the causal mechanism does not take the path through updated infection risk perception, but the causal effect of normative information on *Attendance Change* is significant, we would find a significant Average Direct Effect (ADE).

Another mediator is the infection risk perception after the vignette, which we call *Post-Perception*. This variable measures the respondent’s self-evaluated percentage of getting infected in the next 12 months at the exit of hypothetical scenarios. In short, we posit that a certain threshold of perceived infection risk, lastly updated by the information stimuli in each vignette, would mediate the treatment effects on behavioral changes.

### 3.5. Other variables and sub-sample analysis

Preceding the experimental part, we asked respondents about their gender, age, prefecture they live in, evaluation of the Japanese government’s COVID-19 measures, the number of weddings attended in the past, and trust in the Japanese government, politicians, prefectural governors, and medical experts. We used some of these variables in sub-sample analysis to examine *Exploratory Question 2*. Trust in prefectural governors and medical experts ask the self-reported trust on a 7-point Likert scale, from “Do not trust at all (1)” to “Trust a lot (7).” We also asked seven questions corresponding to the self-deception aspects of the Balanced Inventory of Desirable Responding (Paulhus, 1988) and calculated the factor score (mean = 0, SD = 1.08, See Supplementary File 6 for the detailed operationalization). This score is commonly used in social psychology to identify a potential response bias; the higher the score, the more likely the survey respondents deny psychologically threatening feelings (Paulhus, 1991). While attending marriage during a pandemic is a typical health risk scenario, we expect this score to control potential response bias if any. Gender, age, and prefecture of residence are sampled to be nationally representative based on the latest national census. Following the experimental part, we asked relatively sensitive questions, including the infection experience of participants or anyone in their surroundings, political party support, education level, and income level.

## 4. Results

Table 3 reports the effects of descriptive and injunctive information.

**Table 3:** Effects of information stimuli on *Attendance Change*.

	Model 1	Model 2	Model 3	Model 4	Model 5	Model 6	Model 7	
		Trust in governor		Trust in experts		Self-deception		
		<5	> = 5	<5	> = 5	<0	> = 0	
(Intercept)	0.02	0.04	−0.03	−0.11	0.06	0.16	−0.13	
	(0.06)	(0.07)	(0.10)	(0.09)	(0.08)	(0.10)	(0.07)	
Friends Yes	0.48 ***	0.44 **	0.54 ***	0.52 **	0.46 ***	0.41 **	0.55 ***	
	(0.09)	(0.12)	(0.16)	(0.17)	(0.11)	(0.16)	(0.11)	
Friends No	−0.57 ***	−0.63 *	−0.47 **	−0.41 *	−0.63 ***	−0.70 ***	−0.45 **	
	(0.10)	(0.12)	(0.18)	(0.17)	(0.12)	(0.15)	(0.14)	
Experts Yes	0.12	0.12	0.13	−0.06	0.19	−0.01	0.25 *	
	(0.09)	(0.12)	(0.13)	(0.13)	(0.08)	(0.13)	(0.12)	
Experts No	−0.39 ***	−0.31 **	−0.55 **	−0.12	−0.49 ***	−0.58 ***	−0.20	
	(0.10)	(0.11)	(0.21)	(0.14)	(0.13)	(0.14)	(0.14)	
Friends Yes and Experts Yes	−0.10	−0.20	0.04	−0.23	−0.08	0.06	−0.27	
	(0.14)	(0.18)	(0.23)	(0.25)	(0.17)	(0.21)	(0.18)	
Friends No and Experts No	0.04	0.03	0.05	−0.12	0.11	0.28	−0.20	
	(0.16)	(0.18)	(0.30)	(0.27)	(0.19)	(0.22)	(0.22)	
Friends Yes and Experts No	−0.05	−0.14	0.14	−0.35	0.07	0.09	−0.22	
	(0.15)	(0.17)	(0.27)	(0.26)	(0.18)	(0.22)	(0.19)	
Friends No and Experts Yes	0.20	0.11	0.35	0.17	0.19	0.35	0.06	
	(0.14)	(0.18)	(0.24)	(0.27)	(0.17)	(0.22)	(0.19)	
*N*	1,819	1,150	669	477	1,342	883	936	
R2	0.14	0.14	0.16	0.09	0.17	0.15	0.14	
	Model 8	Model 9	Model 10	Model 11	Model 12	Model 13	Model 14	Model 15
	Age			**Infection risk perception**		**Prefectures**		
	<35	35–60	**>60**	**<=30%**	>30%	**With ED (4)**	Post-ED (6)	**Others (41)**
(Intercept)	0.12	0.02	−0.09	0.00	0.04	−0.03	0.08	0.01
	(0.09)	(0.10)	(0.11)	(0.08)	(0.08)	(0.09)	(0.11)	(0.11)
Friends Yes	0.38 *	0.38 **	0.71 ***	0.58 ***	0.39 **	0.37 **	0.40 **	0.60 **
	(0.19)	(0.14)	(0.19)	(0.14)	(0.13)	(0.13)	(0.15)	(0.18)
Friends No	−1.04 ***	−0.48 **	−0.30	−0.54 ***	−0.61 **	−0.70 ***	−0.49 *	−0.56 ***
	(0.20)	(0.15)	(0.18)	(0.14)	(0.15)	(0.19)	(0.19)	(0.16)
Experts Yes	0.18	0.04	0.24	0.14	0.08	0.22	−0.18	0.23
	(0.18)	(0.13)	(0.18)	(0.12)	(0.13)	(0.14)	(0.15)	(0.16)
Experts No	−0.65 **	−0.33 *	−0.30	−0.22	−0.57 ***	−0.49 **	−0.59 *	−0.56 ***
	(0.23)	(0.14)	(0.20)	(0.14)	(0.14)	(0.17)	(0.24)	(0.16)
Friends Yes and Experts Yes	−0.15	−0.17	−0.49	−0.27	0.08	0.06	0.27	−0.48
	(0.31)	(0.20)	(0.27)	(0.19)	(0.22)	(0.22)	(0.22)	(0.25)
Friends No and Experts No	0.41	−0.21	0.10	−0.15	0.25	0.39	−0.04	−0.10
	(0.36)	(0.22)	(0.29)	(0.21)	(0.23)	(0.29)	(0.34)	(0.23)
Friends Yes and Experts No	0.18	0.02	−0.31	−0.25	0.15	0.16	0.14	−0.30
	(0.32)	(0.21)	(0.28)	(0.20)	(0.21)	(0.23)	(0.33)	(0.23)
Experts Yes and Friends No	0.48	0.08	0.08	0.18	0.23	−0.03	0.31	0.27
	Model 8	Model 9	Model 10	Model 11	Model 12	Model 13	Model 14	Model 15
	Age			**Infection risk perception**		**Prefectures**		
	<35	35–60	**>60**	**<=30%**	>30%	**With ED (4)**	Post-ED (6)	**Others (41)**
	(0.34)	(0.21)	(0.25)	(0.19)	(0.22)	(0.27)	(0.26)	(0.22)
*N*	381	868	570	1,011	807	544	475	800
R2	0.21	0.15	0.11	0.15	0.15	0.18	0.16	0.13

Heteroskedasticity robust standard errors in parenthesis. ***p < 0.001; **p < 0.01; *p < 0.05.

Model 1 in Table 3 supports *Hypotheses 1A, 1B,* and *2B*. Namely, normative information stimuli informing the attitudes of four friends and the official guidance by the prefectural governor and medical experts shift wedding attendance decisions in the expected directions. The only treatment with positive but non-significant effects is *Expert Yes*. Therefore, our findings do not support *Hypothesis 2A*, which predicts the medical experts announcing low risk (*Expert Yes*). We suspect infrequent observation of this kind of official guidance in the actual context resulted in the insignificant effect. Model 1 also reports that all the interaction terms of descriptive and injunctive information are insignificant. A quick answer to *Exploratory Question 1* is to support additive effects. People, on average, examine each injunctive and descriptive information separately and make their decision as assumed by the total of each individual effect.

In the case the two normative information are not aligned, we can conclude that descriptive information surmounts injunctive information when both are present and not aligned since the estimated coefficients of *Friends Yes* and *Friends No* are more substantive than those of *Experts Yes* and *Experts No*. Hence, *Hypothesis 3* is supported. Still, the total additive effect turns insignificant when *Friends Yes* and *Experts No* are provided as information stimuli because of the small margin in beta estimates. When *Friends No* and *Experts Yes* are treated, the total additive effect is negative and significant; people are not likely to attend (see Table 4).

**Table 4:** Attendance Change means and OLS by treatment groups (vignettes).

Treatment groups	*Attendance Change* mean	OLS by treatment group DV: *Attendance Change*
Group 1 (baseline): No expert information + *Friends Do not Know*)	0.02	Baseline group
Group 2 (treatment): No expert information + *Friends Yes*)	0.49	0.48 (0.09)***
Group 3 (treatment): No expert information + *Friends No*)	−0.57	−0.57 (0.10)***
Group 4 (treatment): *Experts No* + *Friends Do not Know*)	−0.37	−0.39 (0.10)***
Group 5 (treatment): *Experts Yes* + *Friends Do not Know*)	0.13	0.12 (0.09)
Group 6 (treatment): *Experts No* + *Friends Yes*)	0.05	0.04 (0.10)
Group 7 (treatment): *Experts Yes* + *Friends Yes*)	0.50	0.49 (0.10)***
Group 8 (treatment): *Experts No* + *Friends No*)	−0.91	−0.92 (0.11)***
Group 9 (treatment): *Experts Yes* + *Friends No*)	−0.24	−0.25 (0.10)*

Heteroskedasticity robust standard errors in parenthesis. ***p < 0.001; *p < 0.05.

Models 2–15 respond to *Exploratory Question 2*, examining the effect heterogeneity among subgroups defined by conditions. In Models 2 and 3, trust in the prefectural governor does not moderate the average treatment effects of the core information stimuli that turned significant in Model 1. In contrast, trust in medical experts matters by turning the effects of *Experts No* insignificant in the low trust group (Models 4 and 5). Those respondents with a higher tendency to deny psychological threats (higher in the self-deception score, whose mean is 0) are not susceptible to *Experts No* stimuli and tend to follow *Experts Yes* (Models 6 and 7). The injunctive and descriptive information that suggests avoiding marriage gathering (*Friends No* and *Experts No*) are not significantly influential to individuals aged 60 or above (Models 8–10). Those who self-reported lower pre-experiment infection risk perception than the median (30%) or equal to it are not susceptible to the *Experts No* stimuli (Models 11 and 12). Lastly, Models 13–15 show the models by the prefectures with emergency declarations at the time of our survey (Tokyo, Chiba, Saitama, and Kanagawa), those with emergency declarations just lifted before the survey started (Osaka, Kyoto, Hyogo, Nagoya, Gifu, and Fukuoka) and the rest. As a result, the distinctive situation in residential prefectures relative to the emergency declaration is not very relevant, except for the higher effect of *Friends No* and the lower effect of *Friends Yes* for those who lived in four prefectures under the emergency declaration when the survey was fielded (Model 13).

Table 5 reports the average causal mediation effects (ACMEs) and the average direct effects (ADEs) of *Perception Change* and *Post-Perception*. We note that aligned or non-aligned combinations of information stimuli do not show influence through the updated infection risk perception. The causal mediation analysis cannot process the interaction terms *per se*, so we have to set one information stimulus at a value and check ACMEs for another information stimulus. Because of this alternative modeling, the average direct effects (ADEs) for two information stimuli in Table 5 are not precisely the same as that of interaction terms in Table 3.

**Table 5:** Average causal mediation effects (ACMEs) and average direct effects (ADEs) by *Perception Change* and *Post-Perception.*

Mediator = *Perception Change*					Mediator = *Post-Perception*				
	Estimate	95% CI Lower	95% CI Upper	value of *p*		Estimate	95% CI Lower	95% CI Upper	value of *p*
*Treated = Friends Yes*									
ACME	−0.00	−0.00	0.00	0.91	ACME	−0.00	−0.01	0.00	0.36
ADE	0.42	0.31	0.55	*p* < 0.001	ADE	0.43	0.31	0.54	*p* < 0.001
*Treated = Friends No*									
ACME	−0.00	−0.01	0.00	0.41	ACME	−0.00	−0.01	0.00	0.45
ADE	−0.49	−0.61	−0.36	*p* < 0.001	ADE	−0.49	−0.62	−0.38	*p* < 0.001
*Treated = Experts Yes*									
ACME	−0.00	−0.01	0.00	0.95	ACME	0.00	−0.00	0.01	0.19
ADE	0.15	0.04	0.27	*p* < 0.01	ADE	0.14	0.03	0.27	*p* < 0.01
*Treated = Experts No*									
ACME	−0.00	−0.02	0.00	0.34	ACME	−0.01	−0.02	0.00	0.02
ADE	−0.39	−0.51	−0.27	*p* < 0.001	ADE	−0.39	−0.51	−0.27	*p* < 0.001
*Treated = Friends Yes, when Experts Yes*									
ACME	−0.00	−0.01	0.00	0.71	ACME	0.01	−0.01	0.02	0.27
ADE	0.35	0.13	0.57	*p* < 0.001	ADE	0.34	−0.13	0.57	*p* < 0.01
*Treated = Friends No, when Experts No*									
ACME	−0.00	−0.02	0.00	0.54	ACME	0.00	−0.01	0.02	0.68
ADE	−0.47	−0.71	−0.21	*p* < 0.001	ADE	−0.46	−0.70	−0.24	*p* < 0.001
*Treated = Friends Yes, when Experts No*									
ACME	−0.00	−0.01	0.01	0.78	ACME	0.00	−0.01	0.02	0.56
ADE	0.40	0.14	0.63	*p* < 0.001	ADE	0.39	0.16	0.61	*p < 0.01*
*Treated = Friends No, when Experts Yes*									
ACME	−0.01	−0.02	0.01	0.4	ACME	−0.01	−0.02	0.01	0.44
ADE	−0.35	−0.57	−0.13	*p* < 0.001	ADE	−0.35	−0.59	−0.11	*p* < 0.01

Quasi-Bayesian confidence intervals, robust standard errors are used.

After all, the ACMEs do not turn significant to whichever two information stimuli are present. The ACMEs of *Perception Change* are insignificant for any informational stimulus. In contrast, the ACMEs of *Post-Perception* are slightly significant for the case in which medical experts alert high infection risk (*Experts No*). Notwithstanding, we note that the proportion of mediated effect is very small (2% on average, 0.04–6% within 95% confidence interval), and the sensitivity analysis tells the mediated effect also depends on other conditions (ACME = 0 at rho = −0.1). We must say that the findings do not support *Hypothesis 4*. Based on our survey, infection risk perception is not a significant mediator when injunctive and descriptive information influences people’s decision-making on behavioral changes.

## 5. Discussion and conclusion

This study has examined the effects of normative information stimuli as a determinant of behavioral changes under the COVID-19 pandemic, with special attention to the interactive effects. Our contributions to the burgeoning literature are threefold. First, we provide experimental evidence by directly testing the interactive effects of injunctive and descriptive information in forming attitudes toward behavioral changes. Scholars have argued about the causal mechanisms regarding citizens’ behavioral changes in Japan without consensus. Official “requests” to adopt infection-preventive behaviors in Japan have been widely broadcasted but never accompanied by legal sanctions. In this ambiguous context, Japanese citizens have widely followed the official guidance, such as maintaining physical distance and wearing masks (Hatabu et al., 2020; Kashima and Zhang, 2021), but why and by what mechanism they do so has been widely debated. Some have emphasized the altruism or sensitivity to the shame that arises when deviating from what is regarded as appropriate behavior by other citizens (Cato et al., 2020; Muto et al., 2020; Nakayachi et al., 2020). However, they are limited to observational data, which does not fully disclose the information process mechanism, and hence is weak as causal inference. Our survey experiment enables a test of multiple information stimuli—aligned and not aligned—in a controlled scenario, reflecting a more realistic information processing context than previous works. Second, we provide the first evidence regarding the synergistic effects of multiple normative stimuli and causal mediation analysis taking infection risk perception as a potential mediator for the effect of information stimuli. Although the findings ratify the supremacy of descriptive norms, not synergistic effects and weak mediation, our systematic test discloses the information processing mechanism and its implications, which is crucial to design strategies for credible science communication under the pandemic. Third, we report that communication by medical experts has limitations but is still effective in specific categories of the population.

Our findings support the individual effects of injunctive and descriptive information (*Hypothesis 1A, 1B, and 2B*) except for *Experts Yes* stimuli (*Hypothesis 2A*), which is unrealistic in the real-world context. The supremacy of descriptive information is also confirmed (*Hypothesis 3*), ratifying the canonical focus theory assumption in social psychology (Cialdini et al., 1990). The infection risk perception is mostly not significant as a causal mediating factor. *Hypothesis 4* is hence not supported. These findings are consistent with the previous literature acknowledging the effects of descriptive norms and, in particular, citizens’ sensitivity to shaming (Cato et al., 2020; Nakayachi et al., 2020). Furthermore, our survey experiment reconfirms this claim with a design explicitly juxtaposing injunctive and descriptive information.

The supremacy of descriptive norms and the absence of synergistic effects have pivotal importance for considering behavioral changes during the pandemic. Even upon facing an uncertain situation like a wedding attendance, people do not seem like processing multiple informational stimuli in a synergistic way, for example, updating normative beliefs due to aligned injunctive and descriptive information. Rather, they seem to judge each information stimulus separately and make decisions accordingly. It makes us suspect of reluctance to admit uncertainty in making behavioral choices within unknown incidence and hence the possibility of normalcy bias, a tendency to minimize the potential threat. If this assumption is valid, scientific communication needs a clear expression to call risk-averse attention. This average response style might be due to the timing of our survey which took place in early 2021, but our survey participants were mostly inexperienced with attending a wedding during the pandemic. The average response style is common across prefectures with and without emergency declaration (Table 3, Models 13–15).

Some intriguing findings are reported around conditioning factors such as trust in medical experts, self-deception bias, age, and baseline infection risk perception (*Exploratory Question 2*). Contrary to claims that young people tend to adopt less risk-averse behaviors than elders (Cucchiarini et al., 2021; Gouin et al., 2021; World Health Organization, 2021), our findings indicate that elders are less prone to following injunctive and descriptive information suggestions to avoid large gatherings (Table 3, Model 10). We quickly add that the actual adherence to social distancing in daily life can be different from what we can guess here from the susceptibility to one-shot information stimuli. Still, it poses an essential question to the previous literature that emphasized young people as a more risk-taking social group (World Health Organization, 2021). This possible anomaly in Japan has been reported partially by Hanibuchi et al. (2021), but we contribute with a more direct analysis involving normative information stimuli presented in a controlled setting.

At the interaction of multiple information stimuli (*Exploratory Question 1*), the findings indicate additive, not synergistic, effects. This result is constant across heterogeneous subgroups. This finding clearly suggests that people care about both injunctive and descriptive information, but it does not mean that they reinforce or update their beliefs relative to infectious deceases instantaneously even when an aligned set of normative information is provided. One may suspect the survey timing was too late to capture a potential synergy under a high uncertainty at the early outbreak of the pandemic. This suspicion might be the case, given that the monthly number of wedding ceremonies was slowly recovering. However, our survey respondents were remarkably divided in their baseline attitudes toward generic wedding attendance during the pandemic (Supplementary Figure S1). Also, our analytical design addresses the pre-post difference in attitudes, controlling the baseline attitudes. Checks on effect heterogeneity keep our findings robust across divergent contexts in age, prefecture of residence, baseline risk perception, and self-deceptive personality.

Our study also highlights that infection risk perception does not explain why people follow experts’ opinions and friends’ behavior. We would say that the ACMEs of *Perception Change* are a conservative test. Whereas the infection risk perception—the mediator—asks a question of a long-time period, the experimental vignette—the independent variable—composes only one of the multiple stimuli people encounter in everyday life. Therefore, it would be unsurprising to see no significant effect from a single stimulus to alter such a long-term vision. The insignificant ACMEs might also be due to the risk perception request format we employed. Meanwhile, it is logically comprehensible that the absolute risk perception level, lastly updated at the exit of the vignette (*Post-Perception*), deters people from attending the wedding. Although this is a tentative conclusion, it opens room for future studies. Like Unkelbach and Speckmann (2021), the repetition of the same message may gradually increase the infection risk perception. As Table 5 suggests partially, it contributes to altering citizens’ behavior when it reaches a certain level. Another room for future study is to diversify scenarios and circumstances to enhance the external validity of our findings.

## Data availability statement

The datasets presented in this study can be found in online repositories. The names of the repository/repositories and accession number(s) can be found below: The dataset and codebook for replication are available on the following web pages. Codebook: https://osf.io/dtzj5 and Dataset: https://osf.io/kewm3.

## Ethics statement

The studies involving human participants were reviewed and approved by the Ritsumeikan University (#衣笠-人-2020-35), Institute of Developing Economies, Japan External Trade Organization (IDE-JETRO), and the University of the Ryukyus (#32). The patients/participants provided their written informed consent to participate in this study.

## Author contributions

IO: conceptualization, methodology, data curation, formal analysis, writing – original draft, visualization, and project administration. IY: funding acquisition, writing, review, and editing. YK and HK: writing – review and editing. All authors contributed to the article and approved the submitted version.

## Funding

This work was supported by the Ritsumeikan University, a research grant by the Research Program for the Post-Corona Society, “Visionaries for the New Normal,” and MEXT/JSPS KAKENHI Grant Number JP20KK0024.

## Conflict of interest

The authors declare that the research was conducted in the absence of any commercial or financial relationships that could be construed as a potential conflict of interest.

## Publisher’s note

All claims expressed in this article are solely those of the authors and do not necessarily represent those of their affiliated organizations, or those of the publisher, the editors and the reviewers. Any product that may be evaluated in this article, or claim that may be made by its manufacturer, is not guaranteed or endorsed by the publisher.

## Footnotes

^1^ https://osf.io/834ma/?view_only=98718b971bac4715be7e5e6a1f6b1c94
