# Supplementary material for: Descriptive, injunctive, or the synergy of both? Experimenting normative information on behavioral changes under the COVID-19 pandemic
Source: Front Psychol. 2022 Dec 22;13:1015742. doi: 10.3389/fpsyg.2022.1015742 (PMC9838221; doi:10.3389/fpsyg.2022.1015742)
Supplement: Supplementary file 1 [file Table_1.DOCX]

Supplementary Material

**Supplementary File 1. Weddings in Japan during the COVID-19 Pandemic**

A marriage ceremony in Japan is usually a social gathering involving 50-90 people, on average, who drink and talk for more than an hour (Kekkon Sougou Ishiki Chousa, 2021). The governments have been ambiguous in their guidance about weddings. A prefectural governor said it is permissible as long as participants do not speak too loudly (Press Conference by the Prefectural Governor of Osaka on July 29, 2020). However, ceremonial hall business associations have alerted the public that some municipalities may restrict wedding events (Regarding the Emergency Declaration, 2021). Empirically, 80-90% of marriage ceremonies were canceled from April to June 2020, then gradually returned to normality (Asahi Shinbun, March 20, 2021). To the best of our knowledge, the only marriage ceremony with a high number of infection cases was reported in Oita prefecture on April 25, 2021, two months after our survey (Yomiuri Shinbun, April 25, 2021). Our survey participants manifested ambiguous perceptions toward wedding participation at the pre-experiment baseline (See S1 Figure).

An average Japanese attends 1 to 20 marriage ceremonies in their entire lifetime, which leads us to predict little chance for survey participants to receive an invitation under the pandemic (Kekkon Sougou Ishiki Chousa, 2021). This feature is advantageous to our study because, while the pre-pandemic blueprint of a marriage ceremony is shared among people, the ‘new normal’ for weddings had not become common knowledge in the first year of the pandemic.

Kekkon Sougou Ishiki Chousa 2021 (結婚総合意識調査 2021). (2021). https://souken.zexy.net/research_news/msgi.html

Regarding the Emergency Declaration (緊急事態宣言の発出に関して). (2021, January 12). https://www.bia.or.jp/bia_info/21-01-12/

# Supplementary File 2. Answer distribution of *Pre-Attendance*


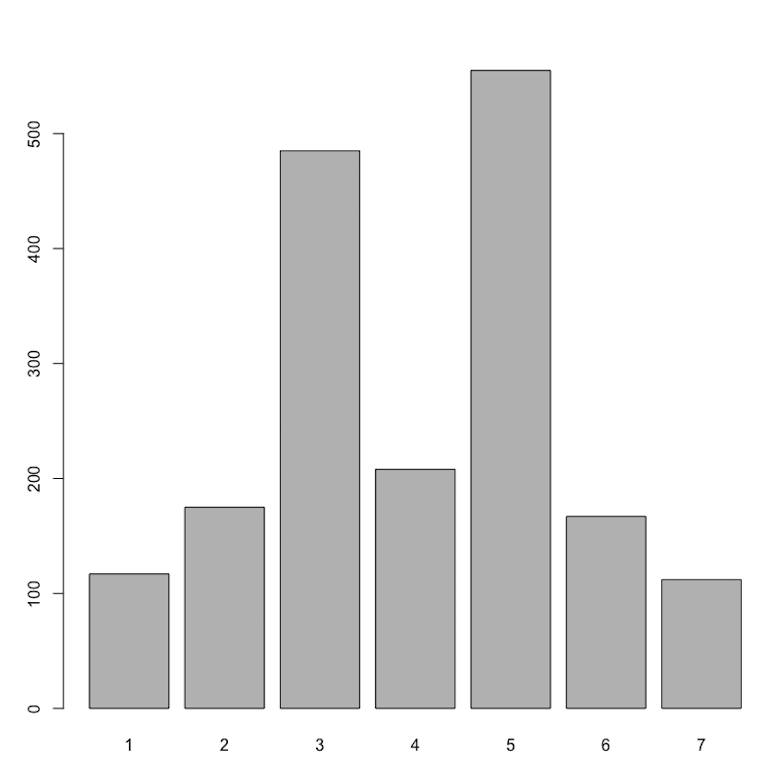


Note: The answer distribution for the question “What do you think of attending a wedding when the infection of novel coronavirus continues? Please imagine that you received an invitation to a wedding from a person who helped you a lot a few years ago, and answer from 7 to 1 when 7 means definitely attending and 1 means definitely not attending.”

# Supplementary File 3. Histogram of *Attendance Change*


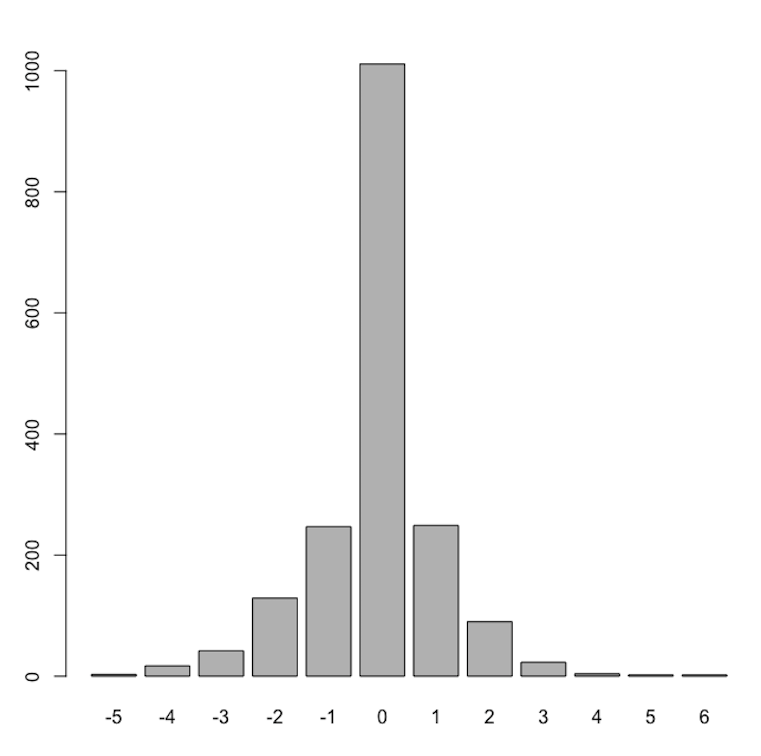


Note: The distribution of margins between Post-Attendance and Pre-Attendance.

# Supplementary File 4. Factual manipulation check

To ensure the effectiveness of information stimuli, we showed six elements and asked participants to mark all they found in the vignette just presented. This task was inserted after the participants answered the questions about dependent variables when they could not look back at the vignette. The table below shows that the information stimuli were effective as we see a high percentage of participants reporting the correct elements included in their respective vignettes. We did not exclude any participants because of this manipulation check.

| Group | Received an invitation | Information about other guests | Opinion of medical experts | Detailed explanation about the ceremonial method | Speech of prefectural governor | Presence or absence of a suggestion from the affiliated organization |  | Either or both of “opinion of medical experts” or “speech of prefectural governor” |
| --- | --- | --- | --- | --- | --- | --- | --- | --- |
| 1 | 86.4% | 82.9% | 6.5% | 59.3% | 3.5% | 61.3% |  | 8.0% |
| 2 | 82.1% | 74.6% | 6.5% | 58.7% | 5.0% | 52.2% |  | 9.0% |
| 3 | 85.2% | 84.2% | 9.4% | 57.1% | 5.9% | 63.1% |  | 12.8% |
| 4 | 84.6% | 70.6% | 42.8% | 59.2% | 56.2% | 64.2% |  | 71.6% |
| 5 | 83.3% | 70.6% | 47.1% | 58.3% | 57.4% | 62.7% |  | 73.5% |
| 6 | 78.0% | 79.5% | 38.5% | 60.5% | 56.6% | 53.2% |  | 66.8% |
| 7 | 84.2% | 72.3% | 51.0% | 60.4% | 51.5% | 50.0% |  | 70.3% |
| 8 | 89.7% | 76.8% | 33.0% | 57.6% | 50.7% | 54.7% |  | 62.6% |
| 9 | 85.1% | 78.1% | 53.2% | 63.2% | 55.7% | 65.2% |  | 70.1% |

# Supplementary File 5. Means and standard deviations by the groups

| Groups | *Attendance Will (prior)* | *Gender* | *Age* | *Income* | *Education* |
| --- | --- | --- | --- | --- | --- |
| Total | 4.012(1.568) | 0.483(0.499) | 49.85(16.19) | 3.26(1.64) | 3.44(1.19) |
| 1 | 4.156(1.531) | 0.535(0.500) | 47.97(16.48) | 3.27(1.51) | 3.47(1.28) |
| 2 | 3.886(1.553) | 0.49(0.501) | 50.80(16.35) | 3.06(1.46) | 3.49(1.12) |
| 3 | 4.163(1.525) | 0.458(0.499) | 49.29(16.92) | 3.11(1.58) | 3.37(1.13) |
| 4 | 4.020(1.546) | 0.46(0.499) | 48.89(15.61) | 3.20(1.61) | 3.68(1.19) |
| 5 | 4.284(1.594) | 0.480(0.501) | 50.75(15.28) | 3.28(1.67) | 3.39(1.18) |
| 6 | 3.912(1.566) | 0.468(0.500) | 48.81(16.52) | 3.28(1.70) | 3.40(1.20) |
| 7 | 3.896(1.582) | 0.532(0.500) | 51.15(16.95) | 3.34(1.68) | 3.46(1.21) |
| 8 | 4.020(1.522) | 0.495(0.501) | 50.13(15.92) | 3.34(1.82) | 3.38(1.19) |
| 9 | 3.856(1.660) | 0.433(0.497) | 50.82(15.60) | 3.48(1.71) | 3.30(1.15) |

Note: standard deviation in parenthesis.

# Supplementary File 6. Factor Analysis: Self-deception

We created the self-deception score by the following steps.

(1) We added seven questions from the self-deception dimension of the Balanced Inventory of Desirable Responding (BIDR) (Paulhus, 1991) that were reported high factor loadings in the previous literature (Tani, 2008) to the questionnaire. The questions were randomly ordered and asked before experimental treatment. The answer option for each question was a 7-point Likert scale, ranging from “not true” (1) to “very true” (7).

(2) Extract a factor score using factor analysis (eigenvalue = 3.12).

**Seven questions**

|  | Original text in BIDR (Paulhus, 1991, translated into Japanese based on Tani, 2008) |
| --- | --- |
| 1 | I never regret my decision. |
| 2 | I am very confident of my judgment. |
| 3 | Once I’ve made up my mind, other people can seldom change my opinion. |
| 4 | I always know why I like things. |
| 5 | It’s all right with me if some people happen to dislike me. |
| 6 | I sometimes lose out on things because I can’t make up my mind soon enough. |
| 7 | I don’t always know the reasons why I do the things I do. |

Paulhus, D. L. (1991). Measurement and Control of Response Bias. In J. P. Robinson, P. R. Shaver, & L. S. Wrightsman (Eds.), Measures of personality and social psychological attitudes (pp. 17–59). Academic Press.

Tani, I. (2008). Balance-gata Shakaiteki Nozomashisa Hannou Shakudo Nihongo-ban (BIDR-J) no Sakusei to Shinraisei Datousei no Kentou. *Personality Kenkyu* 17(1), 18-28.
